# Intravenous Formulation of HET0016 Decreased Human Glioblastoma Growth and Implicated Survival Benefit in Rat Xenograft Models

**DOI:** 10.1038/srep41809

**Published:** 2017-01-31

**Authors:** Meenu Jain, Nipuni-Dhanesha H. Gamage, Meshal Alsulami, Adarsh Shankar, Bhagelu R. Achyut, Kartik Angara, Mohammad H. Rashid, Asm Iskander, Thaiz F. Borin, Zhi Wenbo, Roxan Ara, Meser M. Ali, Iryna Lebedyeva, Wilson B. Chwang, Austin Guo, Hassan Bagher-Ebadian, Ali S. Arbab

**Affiliations:** 1 Tumor Angiogenesis Laboratory, Georgia Cancer Center, Augusta University, Augusta, GA, USA; 2 Cellular and Molecular Imaging Laboratory, Henry Ford Health System, Detroit, MI, USA; 3 Center for Biotechnology and Genomic Medicine, Augusta University, Augusta, GA, USA; 4 Department of Chemistry and Physics, Augusta University, Augusta, GA, USA; 5 Department of Pharmacology, New York Medical College, Valhalla, NY, USA

## Abstract

Glioblastoma (GBM) is a hypervascular primary brain tumor with poor prognosis. HET0016 is a selective CYP450 inhibitor, which has been shown to inhibit angiogenesis and tumor growth. Therefore, to explore novel treatments, we have generated an improved intravenous (IV) formulation of HET0016 with HPßCD and tested in animal models of human and syngeneic GBM. Administration of a single IV dose resulted in 7-fold higher levels of HET0016 in plasma and 3.6-fold higher levels in tumor at 60 min than that in IP route. IV treatment with HPßCD-HET0016 decreased tumor growth, and altered vascular kinetics in early and late treatment groups (p < 0.05). Similar growth inhibition was observed in syngeneic GL261 GBM (p < 0.05). Survival studies using patient derived xenografts of GBM811, showed prolonged survival to 26 weeks in animals treated with focal radiation, in combination with HET0016 and TMZ (p < 0.05). We observed reduced expression of markers of cell proliferation (Ki-67), decreased neovascularization (laminin and αSMA), in addition to inflammation and angiogenesis markers in the treatment group (p < 0.05). Our results indicate that HPßCD-HET0016 is effective in inhibiting tumor growth through decreasing proliferation, and neovascularization. Furthermore, HPßCD-HET0016 significantly prolonged survival in PDX GBM811 model.

Glioblastoma (GBM) is a hypervascular malignant tumor with poor prognosis^1^,^2^. Because of hypervascularity, anti-angiogenic therapies (AATs) targeting the vascular endothelial growth factor (VEGF) and VEGF receptor (VEGFR) axis have been attempted in clinical trials, but the results have not been encouraging^3^. Moreover, our preclinical studies in a rat model of human GBM also showed resistance to the treatment of receptor tyrosine kinase inhibitors (RTKIs) and resulted in paradoxical enhancement of neovascularization and tumor growth^4^,^5^. Therefore, we need an agent that will decrease tumor growth and neovascularization with reduced resistance to therapy.

Recently, studies have shown the role of N-hydroxy-N’-(4-butyl-2 methylphenyl) formamidine (HET0016), a highly selective inhibitor of 20-hydroxy arachidonic acid (20-HETE) synthesis involving enzymes of the CYP4A and CYP4F families, in inhibiting tumor angiogenesis, proliferation, migration, and regulation of CD133+/CD34+EPCs^6^,^7^,^8^. HET0016 was able to inhibit angiogenic responses to several growth factors as well as angiogenesis in gliosarcoma and in the cornea induced by implanted human U251 GBM cells^9^,^10^. Our previous study in breast cancer also showed decreased tumor growth after treatment with HET0016^8^. In previous studies of GBM, HET0016 was prepared in cremophor and DMSO that was administered either orally or intraperitoneally (IP). However, there was limited success in controlling the glioma due to low bioavailability^11^. In the present study, we optimized a condition to make IV formulation of HET0016 with 2-Hydroxypropyl Beta Cyclodextrin (HPßCD) to improve bioavailability and deliver an effective dose of the drug to the tumor site, especially in the hypervascular and hypoxic areas of GBM. HPßCD is a derivative of β-cyclodextrin that has been extensively used as a drug delivery vehicle and recently, FDA has approved the use of HPßCD as a treatment for Niemann Pick Type C disease^12^,^13^. The exploitation of HPßCD as delivery vehicle in GBM may be beneficial due to the porosity of the tumor vasculature, an enhanced permeability and retention (EPR) effect and reduced off target effects in the tumor neovasculature^14^. Moreover, due to smaller size (<1 nm) of the HPßCD encased drug, excess drug can be cleared rapidly through kidneys. Therefore, we believe that the use of HPßCD as a drug delivery system for delivering HET0016 in GBM will not have any detrimental effect.

In the current rat xenograft model of GBM, we have performed magnetic resonance imaging (MRI) studies, which help in analysis of changes in tumor vascular physiology, tumor size, backward transfer constant (k_ep_), tumor plasma volume (v_p_), vascular permeability (forward transfer constant, K^trans^), extravascular and extracellular space volume interstitial space volume fraction (v_e_)^15^. In addition to MRI, we have also evaluated changes in histological parameters, effect on survival and signaling pathways following HET0016 treatment. These parameters may allow for a better understanding of the physiological characteristics of the regional tumor environment and vascularity after HET0016 treatment.

The main purposes of the study are (1) to determine whether HPßCD can be complexed with HET0016 within reasonable period of time (practical kitchen chemistry) to obtain a water soluble formulation that can be administered intravenously, (2) to investigate the effects of IV administration of HET0016 on human and syngeneic GBM tumor growth, and survival in patient derived GBM animal models, vascular parameters, histology, and (3) to identify critical signaling pathways inhibited by HPßCD-HET0016, when administered through IV in the rat GBM xenograft model.

## Results

### IV formulation of HET0016 with 30% cyclodextrin

**Figure 1:**
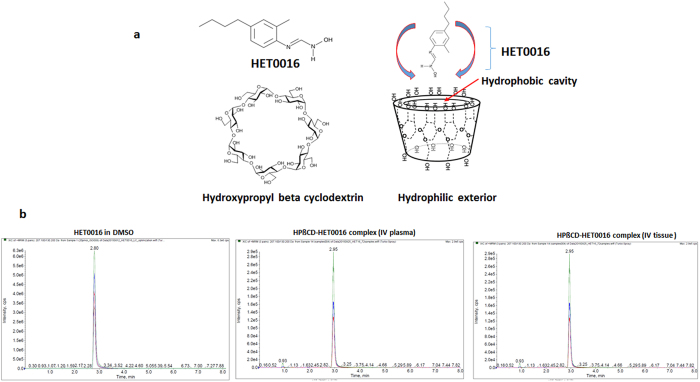
Preparation of HPßCD-HET0016 complex (**a**) Structure of HET0016 and HPßCD is shown. HPßCD resembles a shell and it can encase any deliverable drug. To prepare the IV formulation of HET0016, it was first dissolved in DMSO and added to 30% HPßCD prepared in water. The bucket like structure of HPßCD with hydrophilic exterior helps encase HET0016 and delivers to the target area. (**b**) Mass spectrometry of HET0016 in DMSO (left panel), HET0016 complex in plasma (middle panel). HPßCD-HET0016 complex in tissue lysate (right panel). Plasma and tissue lysate was collected from the rats bearing glioma and injected with single dose of HPßCD-HET0016 for evaluation of pharmacokinetics.

IV formulation of HET0016 using 30% HPβCD (in solution with water) was stable both at 4 °C and room temperature for an extended time without any precipitation of HET0016 (Fig. 1a shows the structure of HET0016 with cyclodextrin). IV administration of 5-day old complex solution did not cause any immediate or late detrimental effect on health of the animals. There was no change in the mass-spectrometric profile between the naked and HPβCD-HET0016 complex alone or mixed with plasma or cell lysate (Fig. 1b). All profiles showed the molecular weight of HET0016 (complex or naked) between 206 and 207 kDa with retention and elution time of 2.9 minutes.

### HET0016 levels in plasma and tumor tissue

**Figure 2:**
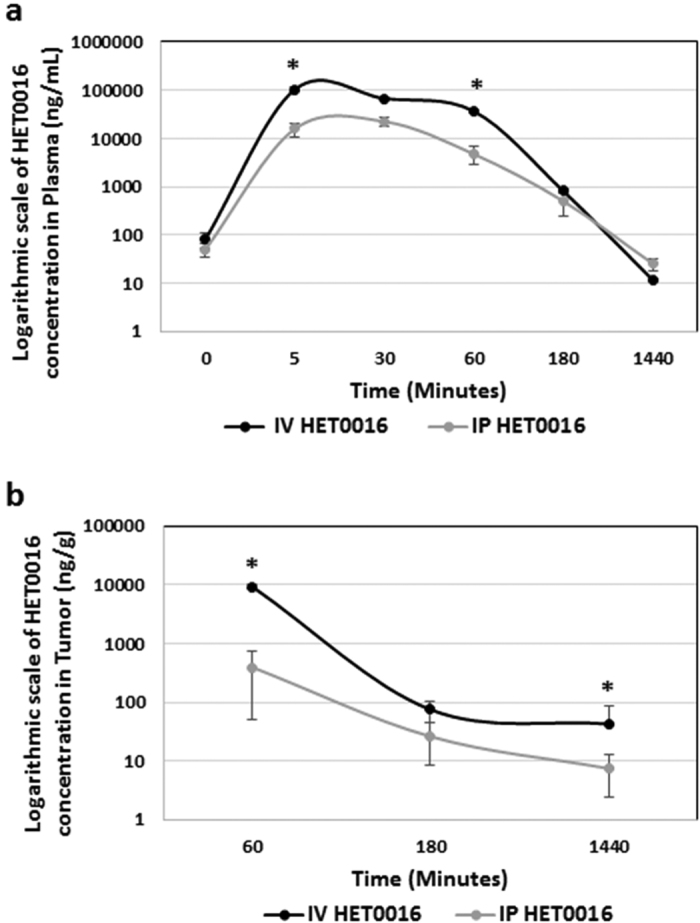
Pharmacokinetics of HPßCD-HET0016 in plasma and tissue after IV and IP dose administration in rat glioblastoma model. The concentrations of HET0016 in plasma or tissue versus time post dose administration in rat model are shown. Blood samples were obtained for 0–24 hrs after dose administration. Plasma and tissue HET0016 concentrations were determined by LC-MS/MS after liquid phase extraction. Each data point is presented as the mean concentration ±SEM (n = 2–4). Concentration of HET0016 in plasma (**a**) and tumor tissue (**b**) after a single HET0016 (10 mg/kg) dose through IV and IP route. Significant values from *t Student* test and Mann-Whitney’s test are represented by *p < 0.05 in comparison to IV and IP doses in the same time point.

Rats were orthotopically implanted with U251 cells and allowed to form the tumor. HET0016 was administered to rats IV and IP as a single dose and sacrificed for pharmacokinetics at different time points. A total of 12 rats were serially euthanized for the measurement of HET0016 levels in plasma and brain tumor tissue at 0, 5, 30, 60, 180 min and 24 h (1440 min) after single IV and IP HET0016 administration (10 mg/kg) (n = 2–4 per time point) and analyzed by LC-MS/MS as described in pharmacokinetics section in material and methods. The level of HET0016 was 7-fold higher in plasma in the group of IV route at 5 min and 60 min time point (105042 and 36880.18 ng/ml) as compared to the IP group (15938.1 and 4773.0 ng/ml) (p < 0.005) (Fig. 2a). The plasma concentration suggests there was rapid elimination of HET0016 with a half-life of approximately 45 min for both the IV and IP groups. The concentration of HET0016 in tumor tissue in the IV group was much higher than that of the IP group at 60 min (9251 ng/g vs 325 ng/g) and 24 h (42.7 ng/g vs 7.4 ng/g) (p < 0.01) (Fig. 2b). There was no significant difference at middle time point at 180 min.

### Effect of HET0016 on tumor growth and vascular parameters

**Figure 3:**
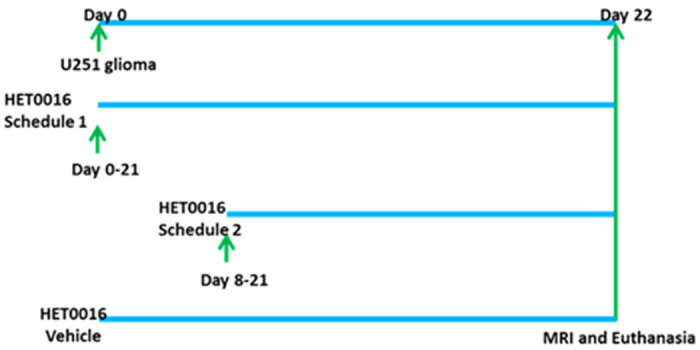
Schematic representation of treatment schedule. U251 glioma cells were implanted orthotopically in rat’s brain and treated with HPßCD**-**HET0016 as described in material and methods.

**Figure 4:**
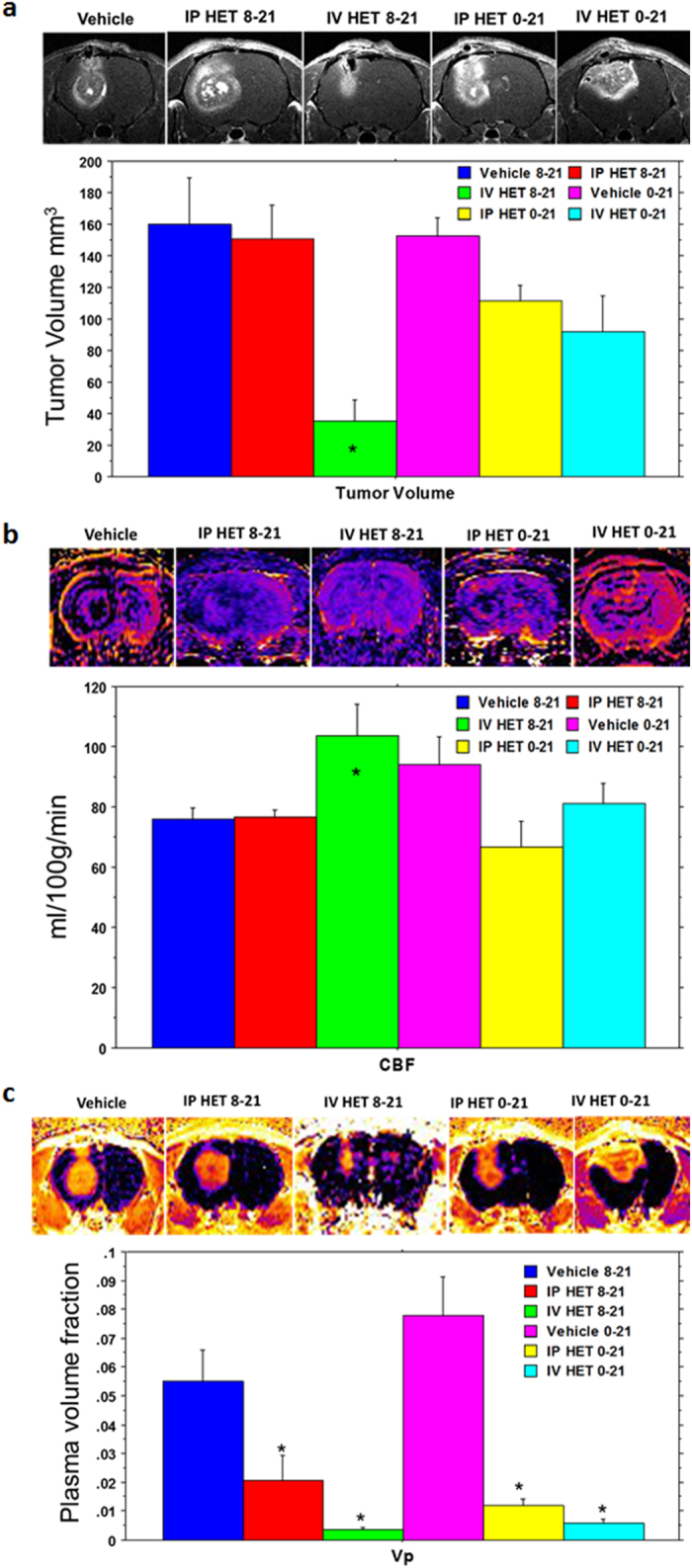
HPßCD-HET0016 reduces tumor growth and vascular parameters in rat glioblastoma model. (**a**) Representative post contrast T1-weighted images from *in vivo* imaging of rat with glioma show tumor size from different groups of animals. Semi-quantitative analysis shows significantly reduced tumor volume in animals that received HET0016 from day 8 and continued for 2 weeks (n = 5 to 8). (**b**) Relative cerebral blood flow maps created from arterial spin labeling techniques. Semi-quantitative analysis (bar graphs) indicates higher flow in IV HET0016-treated groups (started on day 8) compared to that of vehicle-treated group (n = 5 to 8). (**c**) Tumor plasma volume maps and semi quantitative analysis are shown. Both IV and IP treatment with HET0016 caused a significant decrease in tumor plasma volume (v_p_) compared to vehicle and corresponding IP-treated groups. Each group is represented as the mean of the total measurable MRI sections from animals of each group (n = 5–8 animals but 7–16 sections). Significant values from ANOVA test followed by Fisher’s exact test are represented by *p < 0.05 compared to the corresponding vehicle group. An outlier data point (from a single MRI section) was removed as described in our material and methods.

Rats were orthotopically implanted with U251 cells and allowed to form tumor for 8 days. After day 8, animals were treated with HET0016 and followed up for 21 days. A detailed schema of treatment schedule is shown in Fig. 3. Treatments on the same day and seven-day waiting period were chosen to mimic the post-surgical cases and post diagnosis of GBM, respectively, as described previously^4^. Tumor growth was measured by *in vivo* MRI. IV administration of HET0016 significantly reduced the tumor growth (in delayed [day 8–21] treatment) compared to that of vehicle treated animals (p < 0.001) (Fig. 4a) and there was reduced growth with early (day 0–21) treatment although significant difference was not achieved. When compared to the IP administered treatment, tumor growth was reduced only with early IP treatment, although significant difference was not achieved (Fig. 4a). However, when all groups were compared, significant (p < 0.005) reduction of tumor volume was found for delayed IV treatment group compared to the vehicle and delayed IP HET0016 treated groups (Fig. 4a).

**Figure 5:**
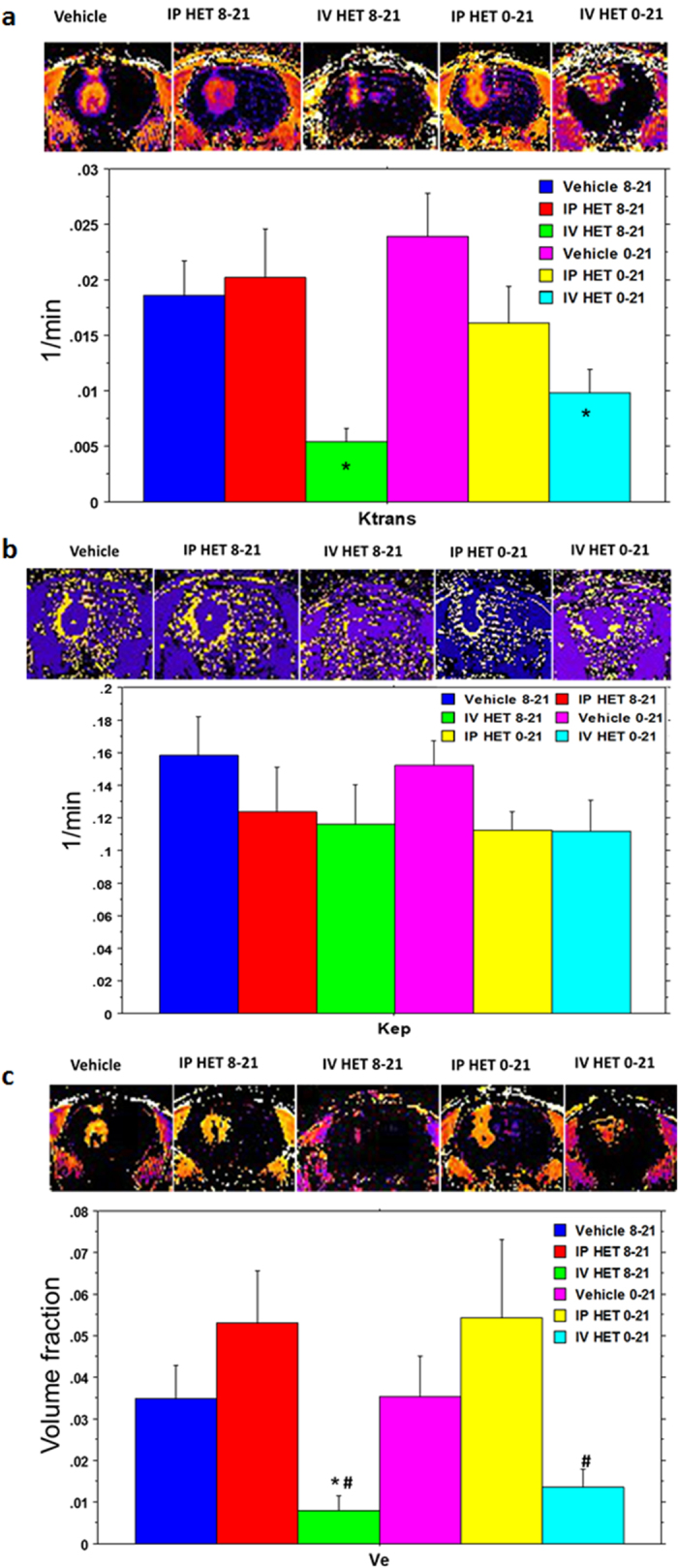
HPßCD-HET0016 influences vascular parameters in rat glioblastoma model. MRI images from *in vivo* rat imaging show significant changes in vessel permeability (K^trans^) (**a**), and v_e_ (interstitial space volume) (**C**) but no changes in k_ep_ (backflow rate constant) (**b**) in early and delayed treatment in IV groups. Significant values from ANOVA test followed by Fisher’s exact test are represented by * and ^#^p < 0.05 compared to (*) vehicle 8–21 vs IV HET 8–21 and vehicle 0–21 vs IV HET 0–21, and (#) IP HET 8–21 vs IV HET 8–21 and IP HET 0–21 vs IV HET 0–21. Each group is represented as the mean of the total measurable MRI sections from animals of each group (n = 5–8 animals but 7–16 sections), analyzed by MRI at 0.5 μm thickness in a range up to 7–16 viewed slices of each animal.

Vascular parameters were evaluated to gain information about the vascular kinetics of the tumor environment. Delayed IV HET0016-treated animals showed significantly increased blood flow, which may indicate normalization of blood vessels causing enhanced flow although there was no significant difference in blood flow (rCBF) among the IP and early IV treated groups (Fig. 4b). As expected, late IP or IV HET0016 treated animals showed significantly lower v_p_ (blood plasma pool), v_e_ (extracellular space or interistial volume), K^trans^ (forward permeability transfer constant) compared to that of corresponding vehicle treated groups (p < 0.01) (Figs 4c and 5a–c). Both early IP and early IV groups also showed significantly lower v_p_ compared to that of corresponding vehicle treated groups (Fig. 4c). As rCBF analysis showed higher blood flow (Fig. 4b), it is expected that normalized vessels will have less K^trans^ (Fig. 5a) compared to that of neovessels. Therefore, the blood plasma pool (v_p_) would be reduced in tumors with normalized vessels (Fig. 4c). When the extravascular extracellular space volume (v_e_) was compared, both early and delayed IV HET0016- treated groups showed significantly decreased v_e_ compared to that of vehicle and IP-treated groups (Fig. 5c). There was no difference in k_ep_ (backflow transfer) between the IP and IV group of animals (Fig. 5b). We also evaluated the blood chemistry to determine the toxicity following 0–21 days of treatment with either vehicle, IV formulated or IP HET0016 in GBM xenografts. We did not observe significant changes on liver function, renal function, important enzymes, pancreas, and lipid profile after HET0016 treatment in IP and IV groups (Supplementary Table 1).

### Proliferation (Ki67), Micro Vessel Density (MVD), Smooth muscle Actin (αSMA), extracellular and extravascular space (EES)

**Figure 6:**
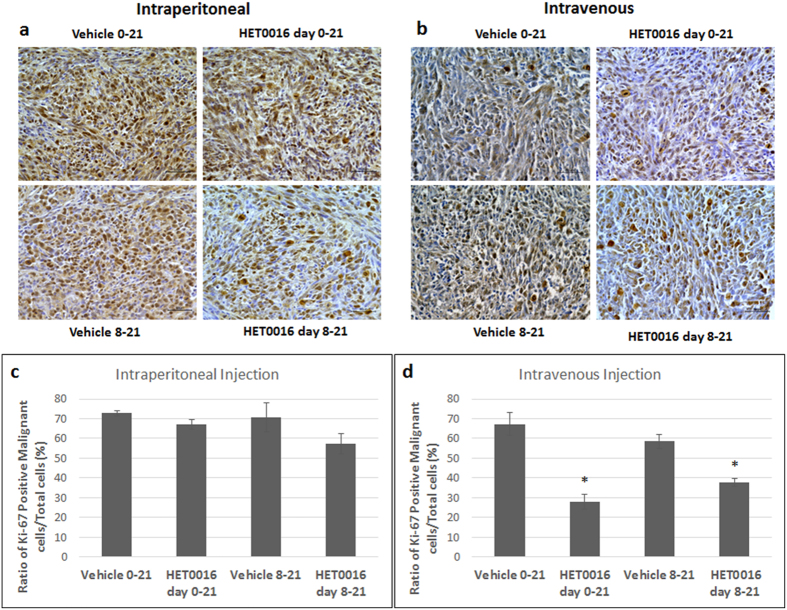
HPßCD-HET0016 treatment results in reduced proliferation, migration in rat glioblastoma model. (**a,b**) Ki-67 immunohistochemical staining was done as a marker of proliferation in tumor tissues. Rats treated with HET0016 starting on day 8 showed significantly fewer proliferative cells in the tumor in the IV group compared to the IP group. (**c,d**) Bar graphs represent the proportion of Ki67 positive tumor cells compared to total number of tumor cells in tumor area of each group. Images were taken in 40x magnification. The brown nuclei color shows the positive labelling cells. Statistically significant differences were verified by ANOVA followed by Bonferroni’s test. *p < 0.05 in comparison to the respective vehicle group.

Brains were removed from the rats (U251 xenograft model) following euthanasia and prepared for immunohistochemistry as described in material and methods. Tumor section in the early and delayed IV or IP treatment and vehicle groups were stained for Ki67, which is a marker of cellular proliferation. Ki67 positive and negative tumor cells were counted within the tumor areas. Ki67 positive cells along the endothelial lining were omitted during counting and calculation of the proliferative cells. In IP treatment groups, only delayed treatment showed reduced proliferation but not significant (Fig. 6a,c). However, in IV treatment groups, significant decreased proliferation was observed in both the early and late treatment groups as compared to corresponding vehicle groups (Fig. 6b–d).

**Figure 7:**
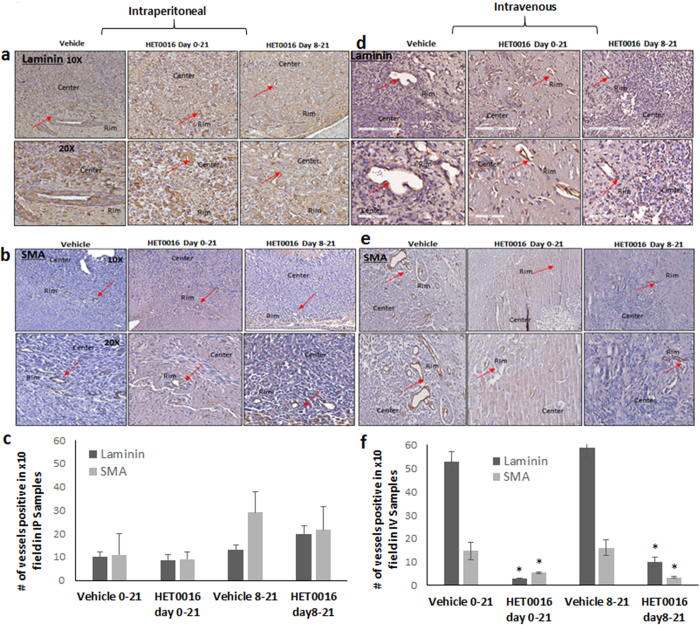
HPßCD-HET0016 treatment results in reduced neovascularization in rat glioblastoma model. Laminin and αSMA immunohistochemistry staining was done in tumor tissues to determine the neovascularization (**a,b,d** and **e**) Representative images from brain tumor tissues are shown at 10x and 20x from the IP (left panel) and IV (right panel) groups. Red arrows indicate blood vessels. Four areas on the tissue section were selected and the number of vessels counted. (**c** and **f**) Laminin and αSMA quanfication. Each bar represents an average of four areas and was estimated in multiple samples from each group (n = 2–4). Significant difference is indicated by *p < 0.05 compared to the respective vehicle group.

Tumor tissues were also stained for laminin to analyze for microvessel density (MVD). Laminin, a basement membrane glycoprotein, is found under the endothelium, encasing the pericytes and smooth muscle cells in the vessel wall and has been shown to be an excellent marker for blood vessels in the brain. Tumor sections were stained with laminin antibody and images from the positive area were taken at 10x and 20x and counted for a number of vessels. We found no significant difference in expression of laminin among the IP-treated groups (Fig. 7a–c, top left panel). However, there was significant reduced expression of laminin with lower number of vessels (3–50 fold less) in the IV treatment group especially around the rim of tumor as compared to vehicle (p < 0.01) (Fig. 7d–f top right panel). We also performed αSMA immunostaining to demonstrate the effect on vessels in pericyte area around tumor periphery and found there was a significantly smaller number of vessels with reduced expression of αSMA in the IV treatment group as compared to vehicle (p < 0.02) (Fig. 7e,f). However, no such differences were detected in the early and delayed IP-treatment groups (Fig. 7b,c).

Differences in the area of extracellular and extravascular space were also analyzed by using H&E stained images from the IP and IV groups using the color thresholding method as described earlier^5^. There was no difference observed among IP treatment groups as compared to vehicle (Supplementary Figure 2a,b top panel). However, we found a threefold reduction in the extracellular and extravascular space in the IV-treated groups (both early and delayed) as compared to vehicle (p < 0.01) (Supplementary Figure 2c,d bottom panel).

### Reduced expression of cell proliferation, inflammatory and angiogenic proteins were detected in glioma treated with HET0016

A protein array was performed using tissue lysates for angiogenic related growth factors using a membrane based protein array kit. Supplementary Figure 3 shows the expression of important proteins related to angiogenesis among the control, early and delayed IV treatment groups. Pro-angiogenic proteins such as VE-cadherin (vascular endothelial cadherin-vasculogenesis), bFGF (basic fibrobalst growth factor), IL-8 (chemokine CXCL8) and MCP-1 (a CCL2 ligand) were significantly downregulated in the delayed IV treatment group while expression of anti-angiogenic proteins such as Tie-2, angiostatin and angiopoietin-2 was increased in the delayed treatment group (day 8–21; Supplementary Figure 3a) as compared to control (p < 0.03). In the early treatment group, expression of proteins, such as MCP-1, angiostatin and angiopoietin-2 followed a similar trend, indicating a significant effect of the treatment, particularly on the factors secreted or expressed by endothelial and inflammatory cells. Western blot was performed to determine the expression of proteins related to cellular proliferation (p-ERK), survival (p-AKT), inflammation (COX-1/2), arachidonic acid metabolism (CYP4A11), signal transducers and activators of transcription (p-STAT1). We also determined the expression of, HIF-1α, EGFR, VEGF, MMP-2 (angiogenesis) and p-NFKB (inflammation), to investigate the effects of IV administered HET0016 on the rat model of GBM. We observed reduced levels of COX-1, CYP4A11, p-ERK, p-AKT, p-STAT1, HIF1α, EGFR, VEGF, MMP-2 and p-NFKB proteins in tumors obtained from both IV treatment groups as compared to vehicle (Supplementary Figure 3b), which confirms the influence of HPβCD-HET0016 on cell proliferation, migration, and inflammation pathways.

### Effect of HET0016 on survival in GBM811 and HF2303

**Figure 8:**
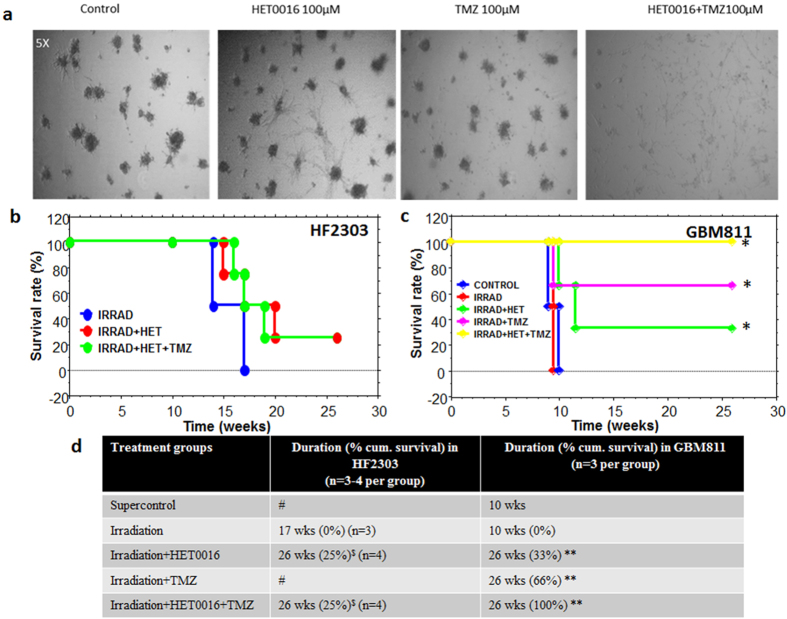
Treatment with HET0016 and TMZ prolongs survival *in PDX models*. (**a**) Treatment with HET0016 and TMZ inhibit neurospheres growth *in vitro:* HF2303 was treated with HET0016 and TMZ alone (100 μM), in combination with TMZ and followed up for 14 days. (**b**) HF2303 and (**c**) GBM811 show the effect of HET0016 and TMZ on survival rate of the mice in groups 1, 2, 3, 4 and 5 of treatment schedules (as described in Material and Methods) were evaluated from the first day of treatment until death. X-axis represents cumulative survival time in weeks. Table in Fig. 8d summarize the details of duration in weeks and % survival. Athymic nude rats were implanted orthotopic with HF2303 and GBM811. Six to ten weeks after implantation, rats were randomized into five treatment groups receiving PBS, radiation, HET0016, TMZ, HET0016 plus TMZ for another 6 wks, as described in Materials and Methods. ^#^The animals were not included due to the technical difficulty. Significant difference is indicated by *p < 0.05 vs irradiation and super-control, ^$^p-value was 0.18 vs irradiation control, achieved by Kaplan Meier analysis and Log-rank test.

We evaluated the effect of HET0016, TMZ alone or in combination with radiation on survival in PDX derived tumor model of GBM811 and HF2303. First, we tested the effect of treatment *in vitro* 3D culture model (HF2303) and found that the treatment with HET0016 or TMZ alone or combination showed the inhibition of the growth of neurospheres Fig. 8a). Similar studies were planned *in vivo* model and we observed that HET0016 treatment alone or in combination with TMZ in irradiated tumor bearing animals resulted in reduced tumor growth and prolonged survival in both GBM811 (*p* < *0.004*) and HF2303 PDX models (p = 0.18) (Fig. 8b). The overall survival in GBM811 model was prolonged to 26 weeks after the treatment with HET0016 plus TMZ and radiation, while control animals (supercontrol and irradiated only control) survived only for 10 weeks similar to the clinical setting (Fig. 8c). The cumulative survival was 100% for HET0016 plus TMZ combination group as compared to 66% for TMZ alone, 33% for HET0016 alone at 26 weeks until the end of the study without demonstrating any evidence of recurrence (Table in Fig. 8d). Therefore, the current study provides evidence that combination therapy (HET0016 + TMZ) with irradiation can improve survival in GBM. In the second PDX model of HF2303, the HET0016 treatment alone and in combination with TMZ, resulted in reduced tumor volume and prolonged survival of 26 weeks vs. only 17 weeks of irradiated control (p < 0.10). The cumulative survival was 25% for both HET0016 plus TMZ combination group and HET0016 alone group at 26 weeks as compared to 0% in radiation only group (p < 0.18). Thus, data indicates combination treatment with HET0016 plus TMZ and irradiation resulted in better survival benefit as compared to radiation alone. Overall, the response was better in GBM811 model than HF2303 model and may be attributed to difference in growth and genetic characteristics. We also measured the presence of 20-HETE and MGMT in different glioma cells (GL261, U251, HF2303 and GBM811) and GBM811 showed comparatively lower amount of 20-HETE and methylguanine DNA methyltransferase (MGMT) levels (Supplementary Figure 5 and 6), which may indicate better treatment results following HET0016, radiation and TMZ in GBM811 PDX model^16^,^17^.

### Effect of IV HET0016 on GL261 syngeneic GBM

We also assessed the effect of IV HET0016 on tumor growth in syngeneic model of GBM using GL261 cell line. All animals underwent MRI and tumor volume was measured from the MRI images. There was significantly reduced tumor volume in animals treated with IV HET0016 (54.33 ± 12.37 mm^3^) compared to that of vehicle treated animals (131.70 ± 19.45 mm^3^) (p < 0.007) (Supplementary Figure 1).

## Discussion

Despite the availability of current chemotherapy and targeted therapies in GBM (e.g. temozolomide and bevacizumab), not many therapeutic benefits have been achieved due to target developing drug resistance^18^. Most therapies have produced a decrease in tumor growth during early stages of treatment followed by aggressive tumor recurrence. Mu *et al*. previously developed water-soluble IV formulation of HET0016 which rapidly penetrated in the rat normal brain and inhibited the formation of 20-HETE after cerebral ischemia^19^. Here, we have developed an improved IV formulation of HPßCD-HET0016 that dissolves in 2–3 min compared to the 48 hrs as reported by Mu *et al*. IV administration of HPßCD-HET0016 in a rat model of human GBM significantly reduced tumor growth in developing or developed tumors as compared to IP HET0016 treatment. It appears that the IV formulation facilitated increased delivery of HET0016 to the hypervascular and hypoxic tumor sites, due to EPR effects. Moreover, the effect of IV HET0016 was not cell specific and showed similar reduced tumor growth in syngeneic tumor (GL261) models. The distribution profile of HPßCD-HET0016 formulation showed better bioavailability and retention in tissue with a higher brain to plasma ratio in the IV treatment group as compared to the treatment with IP preparation. The findings suggest that the IV formulation or encapsulation of HET0016 in a non-toxic delivery system may have the benefits of increased half-life (protect the drug from degradation during circulation and early clearance), lower toxicity, enhanced EPR effect over the IP preparation and improved the therapeutic index^14^. Furthermore, enhanced accumulation of macromolecular drugs in tumor tissues can occur as compared to normal tissue due to impaired lymphatic drainage and porous blood vessels in GBM with abnormal molecular and fluid transport dynamics^20^,^21^. The effect of enhanced delivery of IV HET0016 is supported by vascular parametric analysis in MRI studies. Vascular parametric analysis supported the effect of the drugs, where the IV formulation decreased vascular permeability (K^trans^), tumor blood volume (v_p_), and extracellular extravascular or interstitial space volume (v_e_) but increased the blood flow to the delayed treated tumors in GBM.

TMZ is widely used alkylating agent for the treatment of primary as well as recurrent GBM. Effect of TMZ requires functional DNA mismatch repair (MMR), low levels of methylguanine DNA methyltransferase (MGMT) and DNA repair genes^16^,^22^,^23^. Unfortunately, majority of the primary and recurrent GBMs have unmethylated or active high level of MGMT, which make TMZ treatment unresponsive^17^,^24^,^25^. We evaluated the synergistic effect of HET0016 alone or in combination with TMZ on the survival of animals treated with 30 Gy irradiation in patient-derived xenograft models (PDX) (GBM811, HF2303) (treatment schedule shown in Supplementary scheme). After Administration of HET0016 alone and in combination with TMZ and radiation in GBM811 and HF2303 models for 6 weeks resulted in good response and tumor did not relapse until 6 months (endpoint of the study). Therefore, HET0016 plus TMZ combination may prolong survival and reduce therapy resistance. The synergistic effect may be due to the role of HET0016 in sensitizing the action of TMZ and irradiation by reducing DNA repair mechanisms. Our preliminary study indicated that HET0016 administration resulted in down regulation of DNA repair genes (unpublished preliminary data, Supplementary Fig. 4). The continuous exposure of cellular DNA to potentially harmful environmental and internal insults necessitates redundant and overlapping DNA repair mechanisms^26^. Therefore, successful destruction of GBM tumors may require a combined approach utilizing standard treatments in combination with inhibition of DNA repair pathways.

The anti-tumor effect of HPßCD-HET0016 was supported by decreased tumor cell proliferation, migration, and neovascularization. There was a clear reduction in the number of Ki67 + cells in the IV treatment groups as compared to IP groups. The reason for observing the significant reduced proliferation in IV treatment groups may be that HET0016 is effective in preventing the regrowth of established tumor due to increased bioavailability following IV administration. We also believe established tumor has developed-neovascularization and leaky vessels, which allow EPR effect to be more pronounced in groups treated with IV formulated HET0016. In addition, we suggest that the effect of HET0016 on tumor growth may be attributed to the reduced basic fibroblast growth factor expression (bFGF or FGF-2) after early and delayed treatment. bFGF is a potent mitogen that maintains cancer cell stemness and leads to drug resistance by enhancing the blood-brain-barrier function of endothelial cells^27^,^28^. These results are supported by an earlier study of Guo *et al*.^29^ which showed chronic *in vivo* administration of HET0016 (IP, 10 mg/kg/day) reduced the volume of 9 L gliosarcoma, accompanied by mitotic and vascularization reduction.

GBM tumor vessels are tortuous, disorganized, highly permeable, decreased pericyte coverage and a solid basement membrane structure^30^,^31^. We found overall fewer vessels in both early and delayed IV treatment group, with reduced EES, laminin, and αSMA staining, especially at the invasive margin of the tumor indicating the role of HET0016 in inhibiting the growth of new blood vessels. These observations were supported by a published report that suggested that the pericyte line around new endothelial cells sprouts from tumor vessels, play a role in blood vessel growth, and is suggested to be a potential target in AAT therapy^32^. In addition, this is further supported by a high expression of angiopoietin-2, Tie-2 molecules and reduced VE-cadherin expression, which is associated with pericyte endothelium suggesting an effect of HET0016 on vascular development and stability^33^. We suggest that HPßCD-HET0016 has a primary effect on blood vessels, which by normalization increased the bioavailability of drug to the hypoxic tumor sites. MRI vascular parametric analysis also correlates with immunohistochemistry; especially the normalization of vessels in the IV delayed treatment groups that resulted in decreased permeability (K^trans^) and overall reduced plasma volume fraction (v_p)_. The treatment also caused decreased EES volume indicated by decreased v_e_, which is also validated in H&E histological analysis. Similarly, Guo et al also showed normalization of vessels in 9 L tumor following HET0016 treatment^10^.

In our study, we observed that HET0016 reduced expression of the pro-angiogenic proteins but increased the expression of anti-angiogenic proteins expression to achieve equilibrium to reduce tumor growth. Expression of pro-angiogenic factors such as IL-8, MCP-1, VEGF and SDF-1α were decreased, while expression of inhibitors of the angiogenic process such as angiostatin, angiopoietin-2/Tie-1, and Tie-2 proteins were increased after HET0016 treatment. Recently, a study in triple negative breast cancer showed a similar effect of IP HET0016 on pro-angiogenic factors^8^. We observed that HPßCD-HET0016 reduced IL-8 expression. IL-8 has recently been shown to be a critical factor in regulating cancer cell stemness and invasion, and higher expression was associated with poor survival and therapy resistance in glioma^34^. Moreover, HET0016 treatment reduces the expression of MCP-1 and SDF-1α, key chemokines responsible for trafficking and activation of monocytes/macrophages and have been involved in inflammation and angiogenesis^35^. MCP-1-induced angiogenesis has been reported to be mediated through up-regulation of HIF-1α and subsequent activation of VEGF^35^. Therefore, HPßCD-HET0016 may be acting as an inhibitor of inflammation and angiogenesis growth responses in GBM tumor cells through regulating HIF-1α and VEGF. Interestingly, there were less hypoxic areas in HPßCD-HET0016-treated tumors as shown by reduced HIF-1α protein expression. Reduced HIF-1α expression also resulted in low VEGF expression after HPßCD-HET0016 treatment. VEGF signaling and angiogenesis and highly affected by HIF-1α levels and regulates endothelial cell proliferation, migration, and permeability of blood vessels. Studies have shown high expression is correlated with increased metastases, vasculature, and tumor recurrence^28^. These findings suggest that HPßCD-HET0016 regulated the expression of pro-and anti-angiogenic factors to achieve the balance in the maintenance of the tumor microenvironment^8^.

In the current study, we studied effect of HPßCD-HET0016 treatment in the arachidonic acid metabolism pathway. Previously, HET0016 has been shown to have an effect on expression of CYP4A enzymes. In present study, we found HET0016 was able to reduce the protein expression of CYP4A11 and COX-1, thereby influencing inflammation, angiogenesis, and MAPK signaling in GBM^36^,^37^. Previous studies have also documented a similar role of HET0016 in glioma, gliosarcoma, lung, and breast cancers^10^,^29^,^38^. A study in colon cancer suggested combination therapy of rofecoxib and HET0016 might be a new treatment that can improve the anti-tumor efficacy of rofecoxib alone^39^. In addition, we also investigated role of p-NFκB as marker of inflammation and proliferation. Previous studies have shown a pro-survival role for p-NFκB in glioma and an association with chemo resistance^40^. We demonstrated reduced levels of p-NFκB after HET0016 treatment suggesting an anti-proliferative effect in glioma.

**Figure 9:**
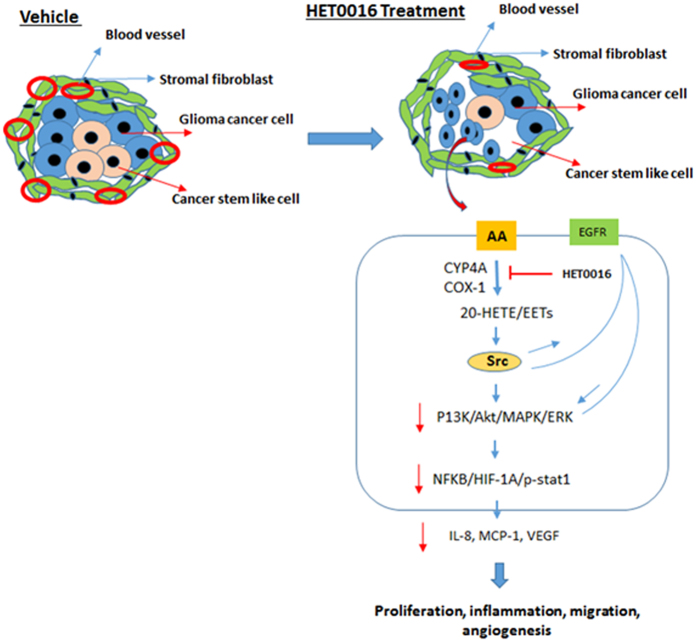
Summary and hypothetical model. Tumors consist of an abnormal vasculature, composed mainly of immature vessels with increased permeability. The less densely packed cells allow drugs or complexes to accumulate in tumor tissue. Treatment with HPßCD-HET0016 leads to reduced expression of αSMA and increased expression of angiopoietin-2 and Tie-2, which may lead to reduced tumor vasculature that is inadequate to support tumor growth and may lead to tumor dormancy. Our results suggest that HET0016 reduces cancer cell growth, invasion, and vasculature by reducing the expression of signaling molecules in the MAPK, PI3K/AKT, and inflammation pathways.

Previous study have shown that GBM has frequent overexpression of EGFR, which leads to activation of the P13/Akt pathway, associated with adverse clinical outcome and has been suggested to be a therapeutic target^41^,^42^,^43^. We showed that HPßCD-HET0016 repressed the expression of EGFR, ERK, and Akt, a target of MEK. We also determined expression of p-STAT1, a tumor suppressor protein involved in the p38/MAPK pathway in response to IFN-α and stress. Higher expression of p-STAT1 was shown to be associated with poor prognosis in glioma and depletion of IRF1/STAT1 signaling has been reported to increase the efficacy of anti-VEGF (bevacizumab) therapy in a glioma xenograft model^44^,^45^,^46^. We found that HPßCD-HET0016 treatment also reduced the levels of pSTAT1, indicating HET0016 can improve overall prognosis in glioma. In summary, we have established a highly soluble HPßCD-HET0016 complex for IV administration that reduced GBM growth, normalized vasculatures, decreased permeability, and EES volume both in established and growing tumors through enhanced bioavailability compared to conventional IP HET0016 preparation. Our results showed that HET0016 in combination with TMZ and radiation enhanced the anti-tumor efficacy and prolonged survival in GBM xenograft models as compared to TMZ alone. Anti-tumor properties of HPßCD-HET0016 resulted in decreasing proliferation, hypoxia, migration, stemness, and vasculatures in glioma by altering the balance of pro-angiogenic and anti-angiogenic balance, PI3K/Akt, p38/MAPK and inflammation pathways (Summary model in Fig. 9). Therefore, HPßCD-HET0016 could be tested in clinics to explore combination therapies with TMZ or radiotherapy for GBM.

## Materials and Methods

### Ethics statement

Animal experiments were performed according to the NIH guidelines and the experimental protocol was approved by the Institutional Animal Care and Use Committee of Henry Ford Health System and Augusta University (formerly Georgia Regents University) (Approval number: 2014–0625). All animals were kept under pathogen-free conditions at room temperature (21 to 25 °C) with exposure to light for 12 hours and 12 hours in the dark. Food and water were offered *ad libitum*. Body weight was measured twice weekly as an indicator of overall animal health. All surgeries were performed under ketamine - xylazine anesthesia, and efforts were made to minimize suffering. Euthanasia for the moribund animals was performed in a CO_2_ chamber.

### Chemicals

HPßCD (2-hydroxy Propyl-β-Cyclodextrin) was purchased from Sigma-Aldrich (St. Louis, MO), cell culture media was from Thermo Scientific (Waltham, MA), and fetal bovine serum was purchased from Hyclone (Logan, Utah). HET0016 was obtained from Dr. JR Falck of UT Southwestern University, Texas, and also synthesized by Dr. Iryna Lebedyeva in the department of Chemistry and Physics, Augusta University with a purity of more than 97%. Cell culture grade DMSO was purchased from Fischer Scientific (PA). Blood chemistry profiles and electrolytes were determined Using third party vendor (Antech Diagnostic).

### Tumor cells

Human glioma U251 cell line was obtained from Dr. Steve Brown of Henry Ford Health System. The cell line was authenticated in July 2014 using the STR profiling method. GL261 syngeneic (C57BL/6 mouse derived) GBM cell line was obtained from Dr. Ted Johnson (Augusta University) and was authenticated in 2016. The cell line, U251 was grown in high glucose (4.5 g/L) Dulbecco’s modified eagles medium (DMEM) and GL261 in RPMI (Roswell Park Memorial Institute) (Thermo Scientific), supplemented with 10% fetal bovine serum (FBS), 2 mM glutamine and 100 U/ml penicillin and streptomycin at 5% CO_2_ at 37 °C in a humidified incubator. Patient derived GBM cells (HF2303) was obtained from Dr. Tom Mikkelsen’ s lab at Henry Ford Hospital and was grown in neurosphere medium (NM), composed of DMEM/F-12 supplemented with N2 (Gibco), 0.5 mg/ml BSA (Sigma), 25 μg/ml gentamicin (Gibco), 0.5% antibiotic/antimycotic (Invitrogen), 20 ng/ml basic fibroblast growth factor, and 20 ng/ml EGF (Peprotech). Cells were maintained in culture for up to passage 10 (low passage). Patient derived PDX GBM cells, GBM811 was obtained from North Western University and was propagated in immunocompromised NOD-SCID mouse and tumor was disintegrated into cell suspension at the time of tumor implantation in nude rats.

### Intraperitoneal (IP) formulation of HET0016

For IP formulation, 2 mg of HET0016 was dissolved in 1 ml of solution containing 80% PBS, 10% of dimethyl sulfoxide (DMSO) (Sigma, St. Louise, MO, USA) and 10% cremophor (Sigma, St. Louise, MO, USA). The mixture was vortexed and sonicated until HET0016 was dissolved. Each rat received 10 mg/kg/day dose and the dose has been used according to previous publications^10^,^11^.

### Intravenous (IV) formulation of HET0016 using HPßCD (2-hydroxy Propyl-B-Cyclodextrin)

Previous published methods have used 48 hours incubation with continuous rotation to make a complex of HET0016 with HPßCD for the IV formulation^19^. Here, we describe a rapid and safe method to synthesize IV formulation of HET0016 without using a sonicated water bath or long rotation. HPßCD has a bucket-like structure with hydrophilic outer shell but hydrophobic inner cavity (Fig. 1a). However, optimized conditions are needed to insert the drug into the HPßCD cage since HET0016 is a heat sensitive compound and can be degraded with long-term rotation at room temperature. HET0016 was dissolved in DMSO (2 mg/50 μl) and was added slowly to a 30% HPßCD solution in sterile water (950 μl) with continuous vortex, and the turbid solution became clear within 2–3 minutes. The final concentration of DMSO was 5%. We have tested mass-spectrometric profiles of HET0016 complexed with HPßCD as well as HET0016 dissolved in DMSO (Fig. 1b). The solution of HET0016 in DMSO showed a peak at 2.9 min retention elution time, and it did not change when HET0016 was complexed or encapsulated with HPßCD.

### HET0016 pharmacokinetics in plasma and tissue lysate

Twelve nude rats (RNU nu/nu) obtained from Charles River Laboratory (Frederick, MD) were used for pharmacokinetics studies. A single IV injection of HET0016 or vehicle (10 mg/kg) in 30% cyclodextrin or IP injection of HET0016 was administered to the animals in the respective groups. Animals were euthanized at multiple time points, and brain tumor tissue was collected (60, 180 min, 24 hrs). The details are provided in Supplementary methods.

### Animal model and treatment schedules

Forty-eight nude rats (RNU nu/nu) weighing 140–150 grams obtained from Charles River Laboratory (Frederick, MD) were used in these experiments. Human glioma U251 cells (400 k in 5 μL), were implanted orthotopically at 3 mm to the right and 1 mm anterior to bregma according to published methods^47^. Following implantation, animals received treatment of HPßCD-HET0016 (IV, 10 mg/kg per day); an equivalent dose of IV HPßCD alone as a vehicle; HET0016 (IP, 10 mgkg/day), or an equivalent dose of IP vehicle alone for either two or three weeks. The details of animal model are described in Supplementary methods.

### *In vivo* MRI and measurement of vascular kinetics

All animals underwent MRI, vascular kinetic, and tumor volume analyses as described previously on day 22^5^ as depicted in Fig. 3. The details on methods are provided in Supplementary methods section.

### Tumor volume analysis

Post contrast T1-weighted images were used to determine the tumor volume. At least two investigators, blinded to the various treatment groups, determined the volume by drawing irregular region of interest (ROIs) for all slices containing tumor. To calculate the exact volume, investigators summed up the number of slices and multiplied by the slice thickness.

### Survival studies in PDX GBM models

Anti-tumor effects and survival studies were conducted in patient derived xenograft GBM model (PDX) using HET0016 as an adjuvant to the current treatment strategies of GBM (radiation and TMZ). Two different PDX models were developed using HF2303 (n = 11) and GBM811 (n = 13). Cells were suspended in PBS and 2 × 10^5^ cells in 5 μl were injected orthotopically into brain of each, 5–7-week-old nude rats, as described previously^47^,^48^,^49^,^50^. Prior to the start of treatment, animals underwent MRI at 6 weeks for GBM811 and 10 weeks for HF2303 before the start of treatment to confirm the presence of a 3 mm^3^ tumor. Thereafter, animals were randomly assigned to five treatment groups and details of the treatment are shown in Supplementary scheme. The five groups of treatment in the present study were as follows: (1) supercontrol; tumor bearing animals were treated with PBS (IV) twice weekly. (2) Irradiation control; tumor bearing animals received a single dose of 30 Gy radiation encompassing the tumor. (3) Irradiation + HET0016; following single dose of 30 Gy irradiation tumor bearing animals received IV HET0016 (10 mg/kg, 5 days/week, every other week for 6 weeks. (4) Irradiation + TMZ; following single dose of 30 Gy irradiation tumor bearing animals received oral TMZ (50 mg/kg, 2 days/week every other week for 6 weeks). (5) Irradiation + HET0016 + TMZ; following single dose of 30 Gy irradiation tumor bearing animals received IV HET0016 plus oral TMZ for six weeks. HET0016 was given on 1^st^, 3^rd^ and 5^th^ weeks and TMZ was given on 2^nd^ 4^th^ and 6^th^ week following irradiation. All animals (GBM 811 and HF2303) received 30 Gy of irradiation in a single fraction before the treatment of TMZ and HET0016 alone or in combination except the super-control group that did not go through irradiation or any other treatments. A single dose of 30 Gy radiation was given in an area encompassing the tumor using an X-ray based image-guided micro small animal radiation research platform from Gulmay Medical Inc. (SARRP^TM^, an Xstrahl company). All surviving animals underwent 2^nd^ set of MRI following 6 weeks of treatments to determine the effects on the tumor volume. 3^rd^ set of MRI was obtained from all surviving animals 4–6 weeks after the end of the treatment. 4^th^ set of MRI was also obtained from all surviving animals at the end of the studies (26 weeks following tumor implantation). All animals were checked for weight gain or loss at least 2 times a week from the day of tumor implantation. Any sign of morbidity was also noted. Survival was calculated from the day of tumor implantation until death. One hundred and eighty-three days after tumor implantation, the experiment was terminated, and the surviving animals were sacrificed. All rats were autopsied at death to examine the antitumor efficacy of each treatment regimen. Our previous experience showed that HF2303 GBM bearing animals die by 16–20 weeks and GBM811 tumor bearing animals die in 12–16 weeks if not treated.

### Histopathology

Tissues were stained for proliferation (anti-Ki 67 antibody, Millipore, USA), human specific MHC-1 marker (anti-MHC-1 antibody, now HLA-A, Abcam), anti-laminin antibody (for blood vessels), and smooth muscle actin (SMA) for pericytes (anti-αSMA antibody abcam, USA) using standard immunohistochemical procedure as described previously and included in Supplementary methods 8.

### Statistical analysis

Comparison between drug and vehicle-treated groups was done by using Student’s *t-test.* When more than two groups were analyzed, we used ANOVA followed by Bonferroni test for normal distribution or Fisher’s exact test. All data were expressed as means ± SEM. *P* value of <0.05 was considered significant value in all tests. The criteria to exclude an outlier were the value outside of the range of mean ± twice the standard deviation (if necessary). Body weight, tumor volume, and survival were compared among PBS-treated, HET0016-treated, TMZ-treated, and HET0016 plus TMZ–treated groups. Kaplan Meier analysis was performed to determine the survival of the animals bearing PDX derived GBM and log rank was used to compare the survival between the two groups.

## Additional Information

**How to cite this article**: Jain, M. *et al*. Intravenous Formulation of HET0016 Decreased Human Glioblastoma Growth and Implicated Survival Benefit in Rat Xenograft Models. *Sci. Rep.* **7**, 41809; doi: 10.1038/srep41809 (2017).

**Publisher's note:** Springer Nature remains neutral with regard to jurisdictional claims in published maps and institutional affiliations.

## Supplementary Material

Supplementary Information
