# Supplementary material for: Intravenous Formulation of HET0016 Decreased Human Glioblastoma Growth and Implicated Survival Benefit in Rat Xenograft Models
Source: Sci Rep. 2017 Jan 31;7:41809. doi: 10.1038/srep41809 (PMC5282583; doi:10.1038/srep41809)
Supplement: Supplementary Information [file srep41809-s1.pdf]

TITLE: Intravenous Formulation of HET0016 Decreased Human Glioblastoma Growth and Implicated Survival Benefit in Rat Xenograft Models

Meenu Jain[1], Nipuni-Dhanesha H Gamage[2], Meshal Alsulami[1], Adarsh Shankar[1], Bhagelu R Achyut[1], Kartik Angara[1], Mohammad Rashid[1], Asm Iskander[1], Thaiz F Borin[1], Zhi Wenbo[3], Roxan Ara[1], Meser M Ali[2], Iryna Lebedyeva [4], Wilson B Chwang [2], Austin Guo[5], Hassan Bagher-Ebadian[2], Ali S Arbab[1]

Supplementary Methods:

HET0016 pharmacokinetics in plasma and tissue lysate

Twelve nude rats (RNU nu/nu) obtained from Charles River Laboratory (Frederick, MD) were used for pharmacokinetics studies. A single IV injection of HET0016 or vehicle (10mg/kg) in 30% cyclodextrin or IP injection of HET0016 (HET0016 dissolved in 80% PBS, 10% cremophor EL and 10% DMSO) or vehicle (80% PBS + 10% DMSO+10% cremophor) was administered, and blood samples were obtained through the tail vein (0, 5, 30, 60, 180 min and 24h). Animals were euthanized at multiple time points, and brain tumor tissue was collected (60, 180 min, and 24h).

HET0016 concentration was determined in plasma and tumor tissue lysate from vehicle and HET0016 treated animals using mass spectrometry. Plasma was collected by centrifuging the blood at 5000 rpm for 10min. Tissue samples were homogenized using 1M phosphate solution containing 1% BSA, 4 M sodium chloride, 10mM EDTA, 0.1% sodium azide and centrifuged at 15,000rpm for 30min at 4ºC. The supernatant (100µl of plasma or tumor lysate) was extracted twice with ethyl acetate (thrice the volume of supernatant); extracts were dried under vacuum evaporator at 37ºc and resuspended in ultra high-pressure (performance) liquid chromatography buffer (UHPLC). Separation of HET0016 samples was performed using a

Phenomenex Kinetex C18 column (100x2.1mm, 1.7um) on a Shimadzu Nexera UHPLC system at a flow rate of 0.2ml/min using an isocratic elution with 51:9:40:0.01 mixture of acetonitrile:methanol:water:acetic acid (UHPLC Buffer) for 8 minutes. The effluent was ionized using positive ion electrospray on an ABSCIEX 4000 QTRAP mass spectrometer with the following instrument settings: ion spray voltage 4500V, curtain gas 30, temperature 450, gas 150, gas 2 50, and low/unit resolution for Q1/Q3. The optimal collision energy, declustering potential, entrance potential and exit potential were determined using purchased standards. The transitions monitored for HET0016 were 207.1-190.3, 207.1-175.1 and 207.1-130.2. The integrated peak areas for these transitions were calculated using Multiquant software (version 2.0, ABSCIEX). Standard curves were generated using eight standards in a range of 0.2 pmol (0.0002 nmol to 2 pmol (0.002 nmol) for HET0016.

Animal model and treatment schedules

Doses were given five days during the week (Monday through Friday). There were two treatment schedules. (1) Treatment was started on the day of tumor implantation with either HET0016 (for IV group n=6, IP group n=8) or vehicle (control group, for IV group n=3, for IP group n=5) and continued for three weeks (0-21 days). 2) Implanted tumors were allowed to grow to 1-3mm in size for 7 days after which the animals received treatments of HET0016 (for IV group n=6, for IP group n=8) or vehicle (control group, for IV group n=6, for IP group n=6) 5 days per week for two weeks (8-21 days). Treatments on the same day and seven-day waiting period were chosen to mimic the post-surgical cases and post diagnosis of GBM, respectively, as described previously[1]. After the study endpoint, animals were euthanized according to IACUC approved protocols and perfused with either ice cold PBS or PBS plus 3% paraformaldehyde. For protein expression analysis, animals were perfused with ice cold PBS and the whole brain

was collected and snap frozen. For immunohistochemistry, animals were perfused with PBS followed by 3% paraformaldehyde. The whole brain was collected and fixed in 3% paraformaldehyde containing 3% sucrose (Fisher Chemical, Waltham, MA, USA).

To determine the effect of IV HET0016 on other GBM, syngeneic GL261 cells (10k/3µl) were orthotopically implanted in immunocompetent C57BL/6 mice (n=12). Six mice received vehicle and six mice received IV HET0016 from day 8 to 21. Animals underwent MRI on day 22.

***In vivo* MRI, vascular kinetic and tumor volume analysis**

All MRI experiments were conducted using a 7 Tesla MRI system (Bruker). Animals were first located with the tumor center corresponding to the magnet center by a tri-planar fast low angle shot (FLASH) sequence. Subsequent scans were acquired using T1-weighted (pre- and post-contrast), T2-weighted, dual gradient echo with a variable flip angle, arterial spin labeling images for creating relative cerebral blood flow (rCBF) maps, and dual gradient echo DCE-MRI scans to estimate K^{trans}, k_{ep}, v_p and v_e. The pre- and -post contrast T1-weighted images were acquired in a spin-echo T1-weighted multi-slice sequence (TR/TE = 800/15.68ms, 128x96 matrix, 27 slices (0.4 mm thick, gap 0.1 mm), number of summed acquisitions (NEX) = 4). Spin-echo T2 weighted images were obtained using standard two-dimensional Fourier transformation (2DFT) multi-slice (27 slices, 0.4 mm thick, gap 0.1 mm) multi-echo sequence: TE/TR = 20,40,60,80/3000, 128x96 matrix, and NEX=2. Prior to the DCE-MRI sequence, and immediately after, two DESPOT1 sequences [2] were run, employing a dual-echo SPGRE sequence (the "mgems" sequence in the Agilent VnmrJ library) allowing a voxel-by-voxel estimation of T_1 in the tissue pre- and post-contrast agent (CA) administration. DESPOT1 sequence parameters are as follows: flip angles = 3, 5, 7, 12, 15, 25, 45 and 70 degrees, matrix

128x64, seven 1.8 mm slices on 2 mm centers, TE/TR=2.0,4.0/80 ms. The DCE-MRI sequence was a dual-echo SPGRE (2GE) sequence with the same geometry and timing as the DESPOT1 sequence, but with a fixed flip angle of 35° and an acquisition of 150 image sets at 5.13 s intervals for a total run time of about 12.8 minutes. At image 15 of the 2GE sequence, a bolus injection of the CA (Magnevist, Bayer Healthcare Pharmaceuticals, Wayne, New Jersey), 0.25mmol/kg at undiluted concentration, no flush, was performed by hand push, followed by a slight draw-back. Prior to DCE MRI and T1 mapping sequences, arterial spin labeling (ASL) images were acquired using FAIR (flow attenuated inversion recovery)-RARE sequence at the center of the tumor to determine rCBF or blood flow through tumor. ASL images were acquired using the following parameters; TR (1) = 12000ms, TE (1) = 46ms, TI (22)= 30 to 2300ms, 128x128 matrix, 32x32 mm FOV, single slice at 1.5 mm thickness, NEX = 1 and bandwidth of 789.14kHz. A Quantitative map was generated using both flow images (on and off images) and T1 maps.

Estimating the vascular parameters using the Patlak Plot graphical method

We have used the DCE MR images and Patlak model to estimate the transfer constants K^{trans}, k_{ep}, plasma volume of tumor blood volume (v_p) and interstitial space volume (v_e) according to the methods previously reported by our group[3]. K^{trans} is the unidirectional transfer rate constant of the contrast from plasma across the vascular endothelium and blood brain barrier (BBB) into the interstitial fluid. k_{ep} is the reverse transfer rate constant from the extravascular compartment to the vascular compartment. v_p is the fractional volume of the contrast agent in the vascular distribution space, usually thought to be the plasma distribution space. If the trans-vascular transfer of the contrast agent is passive, the two rate constants are related via the interstitial space volume fraction: $v_e = K^{trans}/k_{ep}$. Our recent publication shows the validity of this

method[4]. The K^{trans}, k_{ep}, v_p and v_e of the tumors (both treated and control) were determined by drawing irregular ROIs encompassing the whole tumor. At least two investigators blinded to both treated and untreated animals have drawn the ROIs and determine the values. At least two investigators blinded to the treatment groups measured rCBF in the tumors.

Evaluation of proliferation

Ki-67 immunohistochemistry was used to detect the proliferation in the tumor environment. Slides were stained and photographed at 5x, 20x and 40x magnification in five different areas (center, left, right, upper, and lower). Ki67-positive cells (brown) were counted in 40x images from each sample. Images are shown at 40x to show overall staining in the tumor from treatment and vehicle groups.

Evaluation of laminin and SMA staining

Laminin is used to stain lining of blood vessels. and SMA is a validated marker to identify the pericytes in and around the tumor and is up regulated in tumor and inflammation.[5] Tumor tissues were stained with anti-laminin antibody (RB-082-R7) and anti-αSMA antibody (ab5694, 1:100; abcam), photographed in four different areas at 10x and 20x magnifications to demonstrate laminin (vessel) and pericyte- positive areas in tumor.

Evaluation of extravascular extracellular space volume (EES)

Images from H&E stained tumor sections were used to calculate EES volume. Five randomly selected areas were chosen in each section (4 peripheries and 1 central) and photographed at 40x magnification. Areas of extracellular spaces were determined by the color threshold program in image J program (NIH) excluding space within the blood vessels. Data are represented as percent area covered.

Western blot

Protein extraction from vehicle and HET0016-treated tumors was done according to previously described methods[1]. Concentration of protein was determined using BioRad Protein Assay method (BioRad Laboratories). Protein concentrations were estimated with Pierce, BCA protein assay kit (Thermo Scientific, USA), and a total of 50μg protein was separated by standard 4-12% bis-Tris/Glycine/SDS gel electrophoresis. Membranes were probed with primary antibodies for COX-1, CYP4A11 (Abcam; 1:1000), p-AKT, total AKT, p-ERK total ERK, p-STAT1, EGFR, P-NFKB, HIF1A, VEGF (cell signaling; 1:1000), MGMT (Abcam; 1:300), β-actin (Sigma; 1:5000), and horseradish peroxidase-conjugated secondary antibody (1:5000, Biorad).

20-HETE Elisa

Lipid acid extraction from GL261, U251, HF2303 and GBM811 cells were done according to the manufacturer's instructions (Detroit R&D, Inc.). In brief, the samples were collected in triphenylphosphine (TPP) solution (0.05mg/mL), vortexed and sonicated to lyse the cells. The precipitated TPP was separated from the sample solution after centrifugation, homogenized and acidified with acetic acid, then extracted with ethyl acetate for 3 times. The solution was evaporated using nitrogen gas, and resuspended in ethanol and sample dilution buffer provided in the kit. 100 μL of each sample and the standards were loaded in each well, in triplicates. 20-HETE-HPR conjugate was added into the wells and incubated overnight at 4˚C. After washes, TMB substrate was added and incubated for 30min, then neutralized by 2N sulfuric acid and read at 450nm luminescence filter.

Protein Array

Vehicle and HET0016-treated tumor protein (100µg) were collected and processed for protein array analysis. The protein array consisted of 30 proteins that detect angiogenesis, survival, migration and invasion pathways. Analysis was done as described previously[6].

3D culture of HF2303 cells

Formation of neurospheres in 3D culture was tested with vehicle, TMZ alone, HET0016 alone and TMZ+HET0016. Equal number of HF2303 cells was plated in 96-well plate coated with matrigel. After 24 hours, cells were treated with different TMZ or HET0016 alone or in combination, and neurospheres formation was observed for two weeks.

1 Ali, M. M. *et al.* Effects of tyrosine kinase inhibitors and CXCR4 antagonist on tumor growth and angiogenesis in rat glioma model: MRI and protein analysis study. *Translational oncology* **6**, 660-669 (2013).

2 Deoni, S. C., Peters, T. M. & Rutt, B. K. High-resolution T1 and T2 mapping of the brain in a clinically acceptable time with DESPOT1 and DESPOT2. *Magn Reson Med* **53**, 237-241, doi:10.1002/mrm.20314 (2005).

3 Ali, M. M. *et al.* Changes in vascular permeability and expression of different angiogenic factors following anti-angiogenic treatment in rat glioma. *PloS one* **5**, e8727, doi:10.1371/journal.pone.0008727 (2010).

4 Shankar, A. *et al.* Combination of vatalanib and a 20-HETE synthesis inhibitor results in decreased tumor growth in an animal model of human glioma. *OncoTargets and therapy* **9**, 1205-1219, doi:10.2147/OTT.S93790 (2016).

5 Armulik, A., Genove, G. & Betsholtz, C. Pericytes: developmental, physiological, and pathological perspectives, problems, and promises. *Developmental cell* **21**, 193-215, doi:10.1016/j.devcel.2011.07.001 (2011).

6 Borin, T. F. *et al.* HET0016, a Selective Inhibitor of 20-HETE Synthesis, Decreases Pro-Angiogenic Factors and Inhibits Growth of Triple Negative Breast Cancer in Mice. *PLoS One* **9**, e116247, doi:10.1371/journal.pone.0116247 (2014).

Supplementary Scheme: Treatment schedule in PDX model of GBM

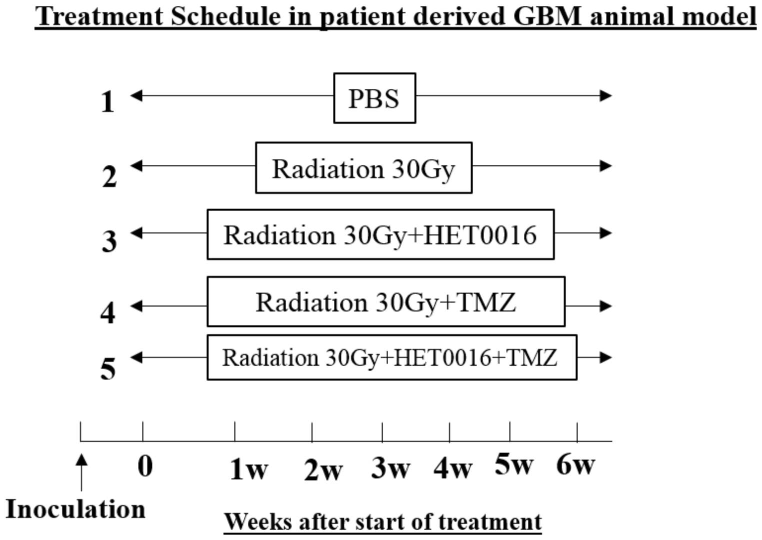

Supplementary Table: Blood biochemistry shows liver (LFT), renal (RFT), lipid, and pancreatic function tests did not show any toxic effect after HET0016 treatment in IP and IV groups.

**Biochemistry Tests**	**Vehicle (Mean± SD) (n=3)**	**IP HET0016 (Mean± SD) (n=3)**	**IV HET0016 (Mean± SD) (n=3)**
**LFT (liver function tests)**			
Total protein	6.367 ± 0.57	6.167 ± 0.32	5.6 ± 0.61
AST (SGOT)	120 ± 74.67	105.33 ± 28.71	91 ± 9.54
ALT (SGPT)	33.33 ± 10.17	39.33 ± 6.11	25.67 ± 0.58
Alkaline phosphatase	105 ± 15	115.67 ± 16.65	73.67 ± 3.22
Total bilirubin	0.100 ± 0	0.100 ± 0	0.100 ± 0
**RFT(renal function test)**			
Bun	16.67 ± 1.53	20.33 ± .58	22.33 ± 5.03
Creatinine	0.367 ± .29	0.267 ± .06	0.267 ± .06
Bun/creatinine	65.33 ± 38.48	79 ± 18.25	89.67 ± 40.51
**LIPID PROFILE**			
Cholesterol	117 ± 34.12	120.33 ± 23.35	84.33 ± 9.87
Triglycerides	82 ± 53.56	50 ± 23.58	50.67 ± 23.16
**ENZYMES**			
Amylase	439.33 ± 86.11	453 ± 92.24	326 ± 23.06
CPK (creatinine phosphokinase)	240.67 ± 116.67	364 ± 161.75	342 ± 141.74
**PSL (pancreatic specific lipase)**	13.67 ± 9.82	9 ± 1	8.67 ± .58

Supplementary Figure 1: Treatment with HET0016 reduces tumor growth in syngeneic GL261 model. Vehicle (n=4) and treatment (n=6). Vehicle and treatment had variable number of mice, as two of the mice in the vehicle group were dead before the end of the study.

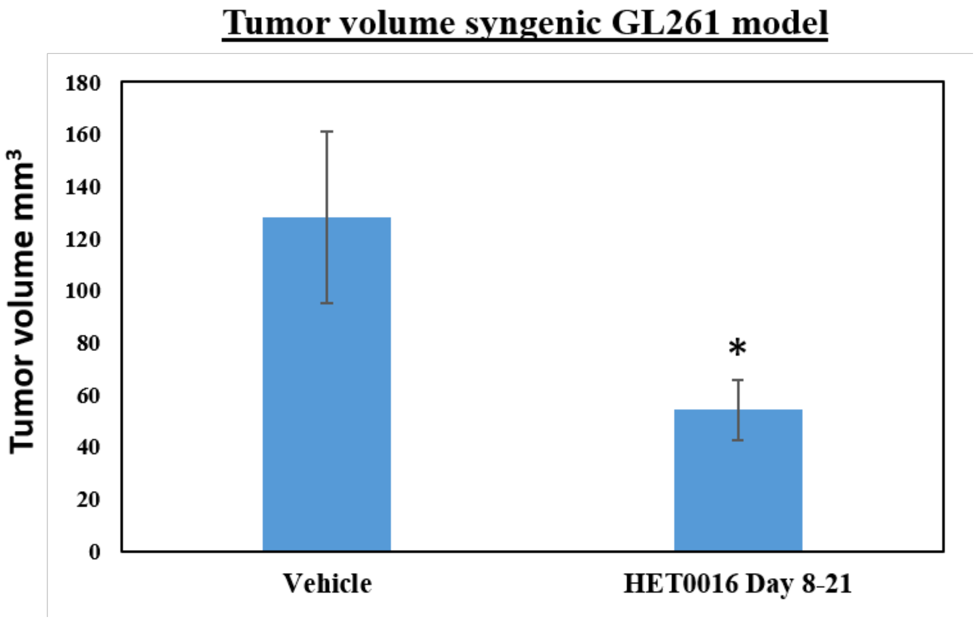

Supplementary Figure 2: HPßCD-HET0016 treatment reduced extravascular extracellular space (EES) in rat glioblastoma model. (A) Formalin fixed slides of tumor tissue from rats were stained with H and E. Images were taken at 10x and 20x in five different areas in IP (top panel), IV group (bottom panel) and EES was analyzed by the color threshold method as described in earlier studies[1]. (B-C) Quantitation of data collected from slides in panel A. Each bar is representative of average of multiple samples (animals) from each group and four areas from each sample (section). Significant difference is indicated by an asterisk (**, *p<0.005; * p<0.05*). We included at least two animals from each group and each animal had 2-3 paraffin blocks bearing tumor tissues.

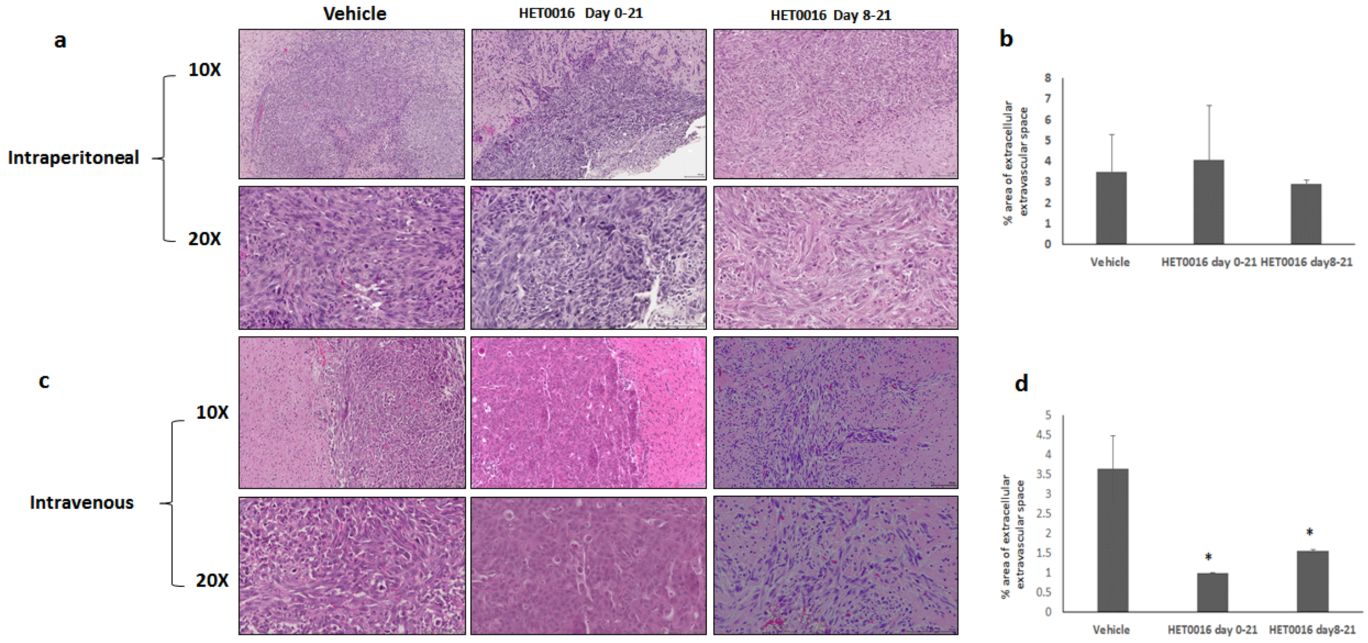

Supplementary Figure 3: Effect of HPßCD-HET0016 treatment on angiogenic growth factors and cell signaling in rat glioblastoma model. (A) Quantitation of selected growth factors and receptors in protein from tumor tissue lysate from different treatment groups using a custom designed membrane-based protein array kit (Human cytokine antibody array kit (RayBiotech, USA)) shows significant differences in various proteins. Anti-angiogenic factors (upper panel), pro-angiogenic factors (middle panel), and cytokines (lower panel) with significant differences in protein levels are shown in the bar graph. Signal intensities were estimated by Image J software. X axis represents signal intensity of protein values normalized to corresponding positive control. B) **HPßCD-HET0016 reduces the expression of proteins involved in arachidonic acid metabolism, MAPK signaling, p-STAT1, HIF-1α in rat glioblastoma model.** Protein lysates from tumor tissues were prepared from animal groups treated with vehicle (30% cyclodextrin) and HET0016 (10mg/kg, IV route) treatment groups using standard RIPA buffer protocol followed by western blot using antibodies as described in methods. Classification of proteins is indicated on the right.

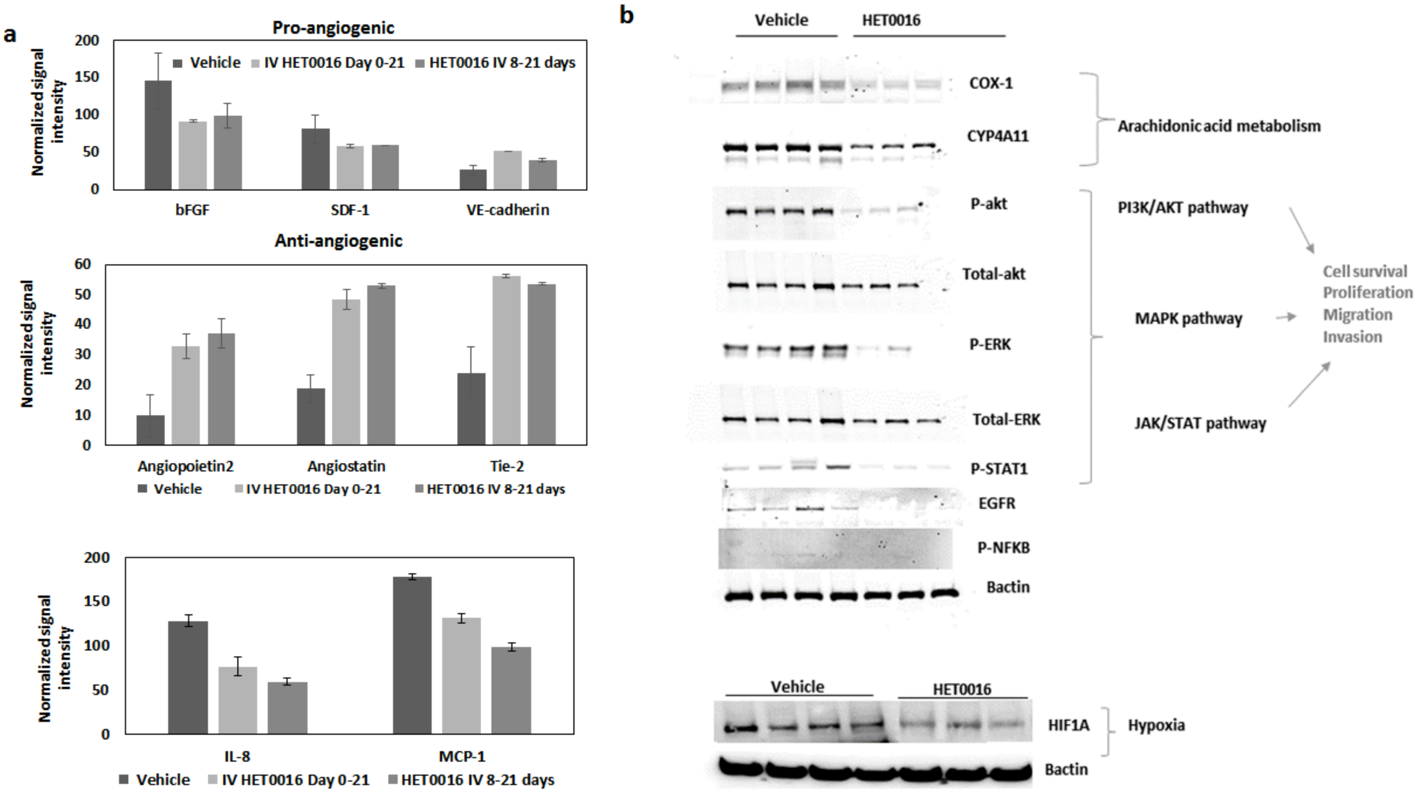

Supplementary Figure 4: Expression of key DNA Repair Genes with greater than 1.5 fold change after HET0016 treatment under normoxia and hypoxia (1%) conditions. U251 glioma cells were treated with 100µM HET0016 for 48hrs. RNA was isolated and gene expression array was performed. Y axis indicates fold change relative to normoxia or hypoxia control. **HET0016 treatment down regulates DNA repair genes involved in NHER, HR, BER, and MMR.** Cells respond to different types of DNA damage by utilizing different DNA repair processes or machineries, including homologous recombination (HR), non-homologous end-joining (NHEJ), nucleotide excision repair (NER), base excision repair (BER) and mismatch repair (MMR). Among them, NHEJ and HR are the two predominant repair pathways for double strand breaks, the most deleterious damage on the chromatin. Therefore, down regulation of repair mechanisms may enhance the effect of HET0016 on DNA damage that ultimately lead to apoptosis and death of cells.

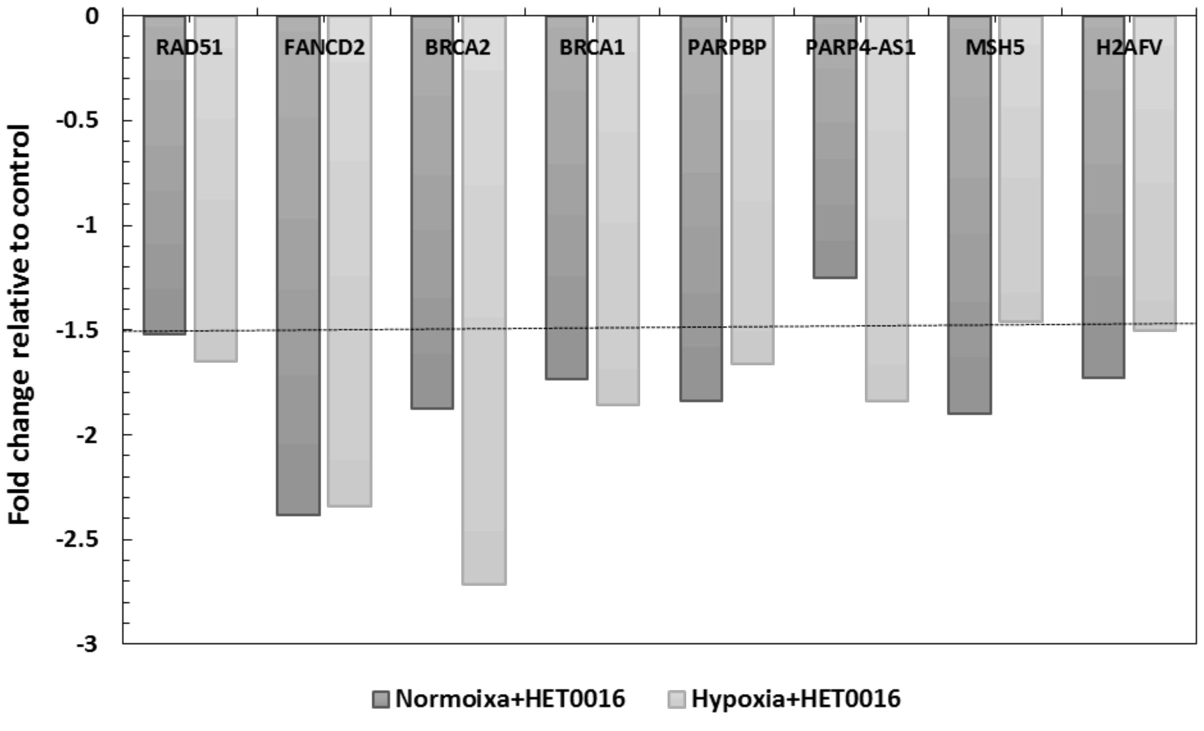

Supplementary Figure 5: Concentration of 20-HETE in cells used for rat and human glioblastoma models, without treatment. Cells were obtained after reaching 80% confluence. ELISA determined 20-HETE concentration after lipophilic acid extraction. Each data point is presented as the mean concentration ± SEM (n=3). Concentration of 20-HETE was normalized by the amount of total protein in the samples.

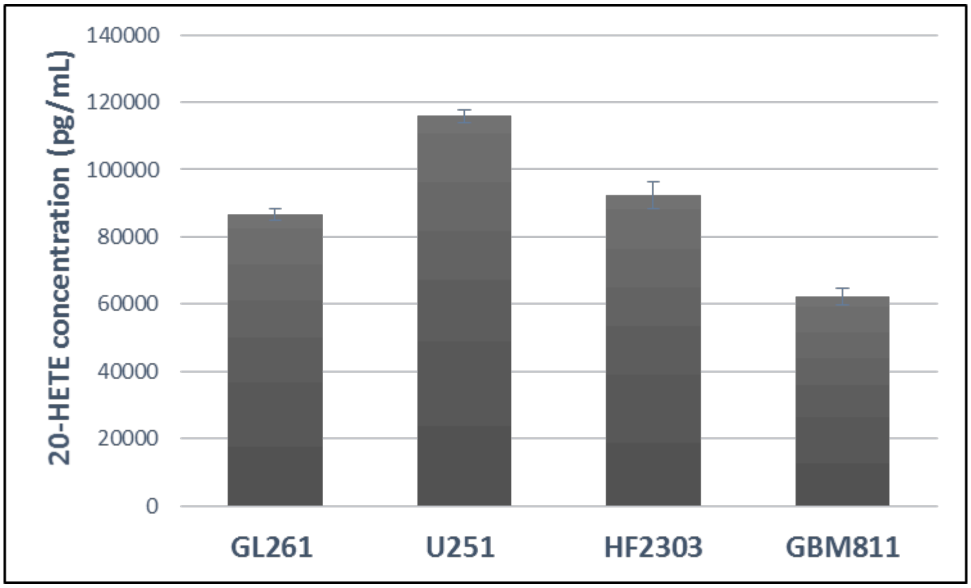

Supplementary Figure 6: Expression of MGMT in cells used for rat and human glioblastoma models, without treatment. Cells were obtained after reaching 80% confluence. MGMT expression was determined by western blotting and normalized by β-actin (signal density). Each data point is presented as the mean percent signal density (compared to corresponding β-actin) ± SEM (n=2).

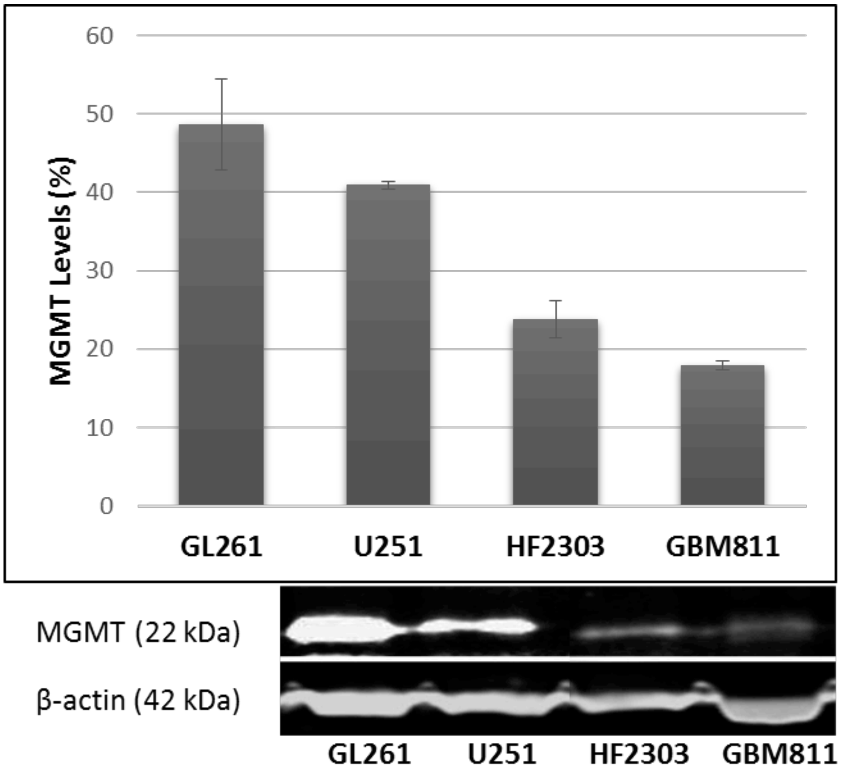

Supplementary Table: Blood biochemistry shows liver (LFT), renal (RFT), lipid and pancreatic function tests did not show any toxic effect after HET0016 treatment in IP and IV groups.

**Biochemistry Tests**	**Vehicle (Mean± SD) (n=3)**	**IP HET0016 (Mean± SD) (n=3)**	**IV HET0016 (Mean± SD) (n=3)**
**LFT (liver function tests)**			
Total protein	6.367 ± 0.57	6.167 ± 0.32	5.6 ± 0.61
AST (SGOT)	120 ± 74.67	105.33 ± 28.71	91 ± 9.54
ALT (SGPT)	33.33 ± 10.17	39.33 ± 6.11	25.67 ± 0.58
Alkaline phosphatase	105 ± 15	115.67 ± 16.65	73.67 ± 3.22
Total bilirubin	0.100 ± 0	0.100 ± 0	0.100 ± 0
**RFT(renal function test)**			
Bun	16.67 ± 1.53	20.33 ± .58	22.33 ± 5.03
Creatinine	0.367 ± .29	0.267 ± .06	0.267 ± .06
Bun/creatinine	65.33 ± 38.48	79 ± 18.25	89.67 ± 40.51
**LIPID PROFILE**			
Cholesterol	117 ± 34.12	120.33 ± 23.35	84.33 ± 9.87
Triglycerides	82 ± 53.56	50 ± 23.58	50.67 ± 23.16
**ENZYMES**			
Amylase	439.33 ± 86.11	453 ± 92.24	326 ± 23.06
CPK (creatinine phosphokinase)	240.67 ± 116.67	364 ± 161.75	342 ± 141.74
**PSL (pancreatic specific lipase)**	13.67 ± 9.82	9 ± 1	8.67 ± .58
